# The holocentricity in the dioecious nutmeg (*Myristica fragrans*) is not based on major satellite repeats

**DOI:** 10.1007/s10577-024-09751-1

**Published:** 2024-05-08

**Authors:** Yi-Tzu Kuo, Jacob Gigi Kurian, Veit Schubert, Jörg Fuchs, Michael Melzer, Ananthu Muraleedharan, Ravi Maruthachalam, Andreas Houben

**Affiliations:** 1 Leibniz Institute of Plant Genetics and Crop Plant Research (IPK) Gatersleben, Corrensstrasse 3, 06466 Seeland, Germany; 2 School of Biology, Indian Institute of Science Education and Research (IISER), Thiruvananthapuram, 695551 Kerala India; 3 Department of Plant Developmental Biology, Max Planck Institute of Plant Breeding Research, 50829 Cologne, Germany

**Keywords:** Centromere type, Dioecious, Holocentric chromosome, Holokinetic, *Myristica fragrans*

## Abstract

Holocentric species are characterized by the presence of centromeres throughout the length of the chromosomes. We confirmed the holocentricity of the dioecious, small chromosome-size species *Myristica fragrans* based on the chromosome-wide distribution of the centromere-specific protein KNL1, α-tubulin fibers, and the cell cycle-dependent histone H3 serine 28 phosphorylation (H3S28ph) mark. Each holocentromere is likely composed of, on average, ten centromere units, but none of the identified and *in situ* hybridized high-copy satellite repeats is centromere-specific. No sex-specific major repeats are present in the high-copy repeat composition of male or female plants, or a significant difference in genome size was detected. Therefore, it is unlikely that *M. fragrans* possesses heteromorphic sex chromosomes.

**Supplementary Information:**

The online version contains supplementary material available at 10.1007/s10577-024-09751-1.

## Introduction

Centromeres are chromosomal regions which determine the faithful segregation of chromosomes during cell division. During mitosis and meiosis, the kinetochore assembles on the centromeres. The spindle microtubules attach to the kinetochore during metaphase, thereby preparing chromosomes for segregation during anaphase. Despite their conserved function among eukaryotes, centromeres are known to possess diverse structural organizations (Schubert et al. 2020). A majority of eukaryotes possess a single, size-restricted centromere per chromosome, manifested as a primary constriction and thus called monocentric. In contrast, species in multiple independent taxa have evolved centromeres throughout the length of the mitotic chromosome and are called holocentric. These chromosomes, which lack a primary constriction, assemble the spindle apparatus on their entire poleward length during mitosis (Hughes-Schrader and Schrader 1961). Unlike monocentric chromosomes, where the migration of centromeres precedes the migration of chromatid arms (as typical V-shaped structures), the sister chromatids of holocentric chromosomes migrate to the poles in parallel (as line-like structures) during anaphase. During interphase, the holocentromeres disperse into multiple centromeric units. While the determination of the centromere type is easy in large-size chromosome species, it is rather challenging in species with small-sized chromosomes, as shown recently in the plant *Prionium serratum* (Baez et al. 2020).

Holocentricity evolved at least 19 times independently in various protozoans, invertebrates, green algae, and higher plant families (Escudero et al. 2016; Melters et al. 2012). Phylogenetically holocentric species are located within broad monocentric clades. Therefore, it is assumed that holocentric species have evolved independently in multiple phylogenetic lineages from monocentric ancestors (Melters et al. 2012). However, the mechanism driving centromere-type transition remains unclear.

In monocentrics, the majority of the centromere-associated DNA are fast-evolving repetitive sequences, including tandem repeats and mobile elements (reviewed in Plohl et al. (2014); Talbert and Henikoff (2020)). In holocentrics, the first centromere-specific repeats were identified in the sedge *Rhynchospora pubera* (Cyperaceae). Each holocentromere of this species harbours several hundreds of regularly spaced 15 to 25 kb-long CENH3-interacting satellite/centromeric retrotransposon arrays (Hofstatter et al. 2022; Marques et al. 2015). A different type of repeat-based holocentromere with an exceptionally high proportion of centromeric satellite DNA (16% of the genome) was identified in the plant *Chionographis japonica* (Kuo et al. 2023). In this species, each of the 69-137 Mb large chromosomes carries 7-11 evenly-spaced CENH3-positive centromere units. Each of the on average ~1.90 Mb large centromere units is composed of 23 and 28 bp-long minisatellites. Thus, different evolutionary pathways may result in various repeat-based holocentromeres (Kuo et al. 2024).

To extend our knowledge of the holocentromere organization of independently evolved holocentrics, we selected *Myristica fragrans* for our study. This dioecious tropical evergreen tree, native to the Maluku islands of Indonesia, is cultivated widely for its seed (the nutmeg) and aril (the mace), both used as a spice or medicine. *M. fragrans* was reported to be holocentric (Ramakrishnan Nair 2019; Flach 1966) through cytological investigation and chromosome fragmentation studies. Due to the small size of the somatic chromosomes, no further conclusive studies using other methods have been carried out to confirm the centromere type of this species.

To ascertain the centromere type of *M. fragrans,* we determined the chromosomal distribution of the conserved centromere-specific protein KNL1 (Oliveira et al. 2024; Neumann et al. 2023), α-tubulin fibers, and the cell cycle-dependent pericentromeric phosphorylation of histone H3 serine 28 (H3S28ph) mark (Goto et al. 2002; Gernand et al. 2003). In holocentric plants, immunolabelling with anti-H3S28ph produces a uniform or line-like staining of condensed chromosomes due to the chromosome-wide distribution of the pericentromere (Gernand et al. 2003; Kuo et al. 2023). Based on the chromosomal distribution of KNL1 and H3S28ph, we conclude that *M. fragrans* is a holocentric species. However, none of the identified high-copy satellite repeats showed typical distribution pattern of holocentromeres. Thus, *M. fragrans* might possess mobile element-based centromere units, or the holocentromere of *M. fragrans* is only epigenetically defined. Furthermore, we found no significant differences between the high-copy repeat compositions of male and female *M. fragrans* in our comparative repeatome analysis.

## Materials and methods

### Plant materials

Fresh seeds of *Myristica fragrans* (Houtt.) were germinated in a dark, humid chamber at 28°C. The seedlings were replanted once they started shoot growth. The young plants were grown in greenhouse conditions, 16 h light (from 6 a.m. to 10 p.m.), day temperature 22°C, and night temperature 18°C. The meristems of roots from young seedlings were used to prepare chromosome slides for immunostaining and *in situ* hybridization experiments.

### Genome size determination by flow cytometry

To isolate nuclei, approximately 0.5 cm^2^ of fresh leaf tissue from *M. fragrans* and the internal reference standard, *Lycopersicon esculentum* Mill. convar. infiniens Lehm. var. flammatum Lehm., Stupicke Rane, Genebank accession number LYC 418, were chopped together in a petri dish using the reagent kit ‘CyStain PI Absolute P’ (Sysmex-Partec) following the manufacturer’s instructions. The nuclei suspension was filtered through a 50-μm CellTrics filter (Sysmex-Partec) and measured on a CyFlow Space flow cytometer (Partec-Sysmex). At least five independent measurements were performed of each of the four individual plants. The absolute DNA content (pg/2C) was calculated based on the values of the G1 peak means and converted to the corresponding genome size (Mbp/1C) according to Dolezel et al. (2007).

### Isolation of genomic DNA and genome sequencing

The sex of *M. fragrans* plants was determined according to the morphological characteristics of their flowers. Genomic DNA was extracted from leaf tissue using the CTAB protocol (https://opsdiagnostics.com/notes/protocols/ctab_protocol_for_plants.htm). Low-pass paired-end (PE, 2 × 150 bp) genome sequencing was performed using the Illumina NovaSeq6000 system by Novogene (UK).

### *In silico* analysis of the repeatome

The quality of genomic Illumina reads of the male and female plants of *M. fragrans* was assessed by FastQC (Andrews 2010) implemented at the RepeatExplorer Galaxy server (https://repeatexplorer-elixir.cerit-sc.cz/galaxy/) and filtered by quality with 95% of bases equal to or above the cut-off value of 10. Qualified paired-end (PE) reads equivalent to ~0.22× genome coverage were randomly sampled and applied for genome repetitive analysis by a graph-based clustering method using RepeatExplorer2 pipeline (Novák et al. 2020), with the default setting of 90% similarity over 55% of the read length. The automatic annotation of repeat clusters was manually inspected and revised if necessary, and the organelle clusters were discarded, followed by a recalculation of the genome proportion of each repeat type. The comparative clustering analysis was performed using one million randomly sampled reads from the male and female samples each.

### FISH probe preparation

The consensus sequences of putative satellites reconstructed by TAREAN (TAndem REpeat ANalyzer) (Novak et al. 2017) were used to design oligo probes. The fluorochrome-conjugated oligos, 5´-FAM- ATCTTGTTGAACCATTTGATTGGTTTGAA-3’ and 5´-TAMRA- GTAATATATGTTTTCGGGGTAGCTCGGAG-3’ were synthesized and modified by Eurofins (Germany), and were used to detect the satellite repeats MfSat269 and MfSat351, respectively. The clone pAtT4 (Richards and Ausubel 1988) was used as the probe to detect the *Arabidopsis*-type telomere. The plasmid DNA from the clone was labeled with ATTO488-dUTP using a Nick Translation Labeling kit (Jena Bioscience, Germany).

### Indirect immunodetection

To prepare mitotic chromosomes and interphase nuclei, root tips and young shoots were first pretreated in 2 mM 8-hydroxyquinoline at 20°C for 4 h. The pretreated material was fixed in 4% paraformaldehyde with 1% Igepal (Sigma-Aldrich) in Tris buffer (10 mM Tris, 10 mM EDTA, 100 mM NaCl, 0.1% (v/v) Triton X-100, pH 7.5) for 5 min under vacuum, followed by another 30 min on ice. The fixed tissue was washed twice in Tris buffer for 5 min, and incubated in an enzyme mixture (0.7% Cellulase Onozuka R10 (Duchefa Biochemie, cat. no. C8001), 0.7% CELLULYSIN^®^ Cellulase (Calbiochem, cat. no. 219466), 1.0% Pectolyase (Sigma, cat. no. 45-P3026), 1.0% Cytohelicase (Sigma, cat. no. C8247)) at 37°C for 45-60 min. The enzyme-treated meristems were chopped in LB01 nuclei isolation buffer (15 mM Tris, 2 mM Na_2_EDTA, 0.5 mM spermine, 80 mM KCl, 20 mM NaCl, 15 mM β-mercaptoethanol, and 0.1% (v/v) Triton X-100), and filtered through a 50-μm CellTrics filter (Sysmex-Partec). Cell suspensions were centrifuged onto Superfrost Plus Adhesion Microscope slides (Epredia) using a Cytospin3 centrifuge at 700 rpm for 5 min. The primary antibodies diluted in 2% BSA in 1×PBS buffer with 0.5% (v/v) Triton X-100 and 0.2% (v/v) Igepal (Sigma-Aldrich) were applied onto the slides, followed by incubation at 37°C for 1 h, and afterwards at 4°C overnight. Before washing, the slides were incubated at 37°C for 1 h and subsequently washed twice in 1×PBS buffer for 5 min, followed by 1 h incubation at 37°C with the secondary antibody diluted in 1% BSA in 1×PBS buffer with 0.5% (v/v) Triton X-100 and 0.2% (v/v) Igepal (Sigma-Aldrich). After washing twice in 1×PBS buffer, the slides were dehydrated using a 70, 90, and 100% ethanol series for 2 min each, followed by air drying and counterstained with 10 μg/ml 4′,6-diamidino-2-phenylindole (DAPI) in Vectashield Antifade Mounting Medium (Vector Laboratories).

The primary antibodies used in the study were rabbit anti-*Cuscuta europeae* KNL1 (diluted 1:400) (Neumann et al. 2023; Oliveira et al. 2024) and the commercially available antibodies rat anti-histone H3S28ph (Sigma Aldrich, cat. No. H9908-2ML, diluted 1:1000) and mouse anti-α-tubulin (Sigma, cat. No. T9026-2ML, diluted 1:300). For immunodetection of microtubules, the pretreatment with 2 mM 8-hydroxyquinoline was excluded, and the Tris buffer and 1×PBS buffer mentioned above were substituted by 1× Microtubule Stabilizing Buffer (MTSB) (50 mM PIPES, 5 mM EGTA, 5 mM MgSO_4_, pH 6.9). Anti-rabbit rhodamine (Jackson ImmunoResearch, cat. no. 111295-144, diluted 1:300), anti-mouse Alexa488 (Jackson ImmunoResearch, cat. no. 715-546-151, diluted 1:300), and anti-rat Alexa488 (Jackson ImmunoResearch, cat. no. 112-545-167, diluted 1:300) were used as secondary antibodies.

### Fluorescence *in situ* hybridization

Flower buds collected from male *M. fragrans* plants were fixed in 3:1 (ethanol: glacial acetic acid) fixative and were used to prepare chromosome spreads. Fixed anthers were dissected from flower buds and washed in 1× citrate buffer (0.01 M sodium citrate and 0.01 M citric acid, pH 4.5) for 5 min. To overcome the dense cytoplasm in *M. fragrans* cells, anthers were pretreated with 1% Triton X-100 and 2% (w/v) PVP dissolved in 1× citrate buffer for 10 min, according to Lunerová and Vozárová (2023). The anthers were then washed in 1× citrate buffer for 5 min and incubated in an enzyme mixture (0.7% Cellulase Onozuka R10 (Duchefa Biochemie, cat. no. C8001), 0.7% CELLULYSIN^®^ Cellulase (CalBiochem, cat. no. 219466), 1.0% Pectolyase (Sigma, cat. no. 45-P3026), 1.0% Cytohelicase (Sigma, cat. no. C8247)) at 37°C for 2 h. The treated anthers were ground in 45% acetic acid. The cell suspension was mixed with acetocarmine on a microscope slide and squashed under a coverslip. The coverslip was removed after freezing the slide in liquid nitrogen, and the slides were air-dried.

Before hybridization, the slides were pretreated with 45% acetic acid at room temperature, followed by 0.1% pepsin in 0.01 N HCl at 37°C, and postfixed in 4% formaldehyde at room temperature for 10 min each. Each of the three pretreatment steps was followed by washing twice in 2×SSC for 5 min. The slides were dehydrated in a 70, 90 and 100% ethanol series for 3 min each and air-dried.

Probes were denatured at 95°C for 10 min in hybridization mixture (50% (v/v) formamide, 10% (w/v) dextran sulfate, 2×SSC and 5 ng/µl of each probe) and kept on ice until use. For 20 µl hybridization solution, 1 µl of each probe was used and the rest was filled up by the hybridization mixture. Hybridization solution was applied on dry slides and covered with a coverslip. Slides were denatured at 75 °C for 2 min on a hot plate and were incubated at 37°C overnight. Coverslips were removed in 2×SSC, and slides were washed in 2×SSC at 57°C for 20 min in a water bath, followed by dehydration in a 70, 90 and 100% ethanol series, air-dried, and counterstained with 10 μg/ml DAPI in Vectashield Antifade Mounting Medium.

### Immuno-FISH

After removing the coverslip and washing away the DAPI-containing mounting medium with 1×PBS, the immunostained slides were postfixed in 3:1 (ethanol-glacial acetic acid) fixative at room temperature for 10 min, and directly dried in darkness. Afterwards, the slides were pre-hybridized with the hybridization mixture mentioned above at 37°C overnight, in a humid chamber. The slides were washed in 2×SSC for 5 min and dehydrated in a 70, 90 and 100% ethanol series for 3 min each. Denaturation was performed in 0.2 N NaOH in 70% ethanol for 10 min at room temperature. The incubated slides were washed in ice-cold 1×PBS for 1 min, dehydrated in a 70, 90 and 100% ethanol series and air-dried. Probe denaturation, hybridization, and counterstain were performed as described above; only the stringent wash was carried out at room temperature.

### Microscopy and image analysis

To analyze the chromatin at the ultrastructural level, we applied super-resolution spatial structured illumination microscopy (3D-SIM) using a 63x/1.40 Oil Plan-Apochromat objective of an Elyra PS.1 microscope system (Carl Zeiss GmbH). Image stacks were captured separately for each fluorochrome. Maximum intensity projections from image stacks were calculated using the Zeiss ZENBlack software. Zoom-in sections were presented as single slices to indicate the chromatin structures at the super-resolution level (Weisshart et al. 2016). To visualize the spatial chromatin organization and localization of FISH- and immuno-signals, the Imaris 9.7 software (Bitplane) was applied to render 3D image stacks. The number of KNL1 foci in interphase nuclei was determined using the Imaris tool ‘Spots’ (Randall et al. 2022).

### Transmission electron microscopy

To avoid fixation artifacts and to achieve the best possible structure preservation, cuttings of 2 mm^2^ from the central part of mature leaves of *M. fragrans* plants were used for high pressure freezing (HPF) with a Wohlwend high pressure freezing machine HPF Compact 03 (Wohlwend GmbH, Switzerland). HPF, cryosubstitution, resin embedding, sectioning, and ultrastructure analysis by transmission electron microscopy were performed as described (Daghma et al. 2011).

## Results

### *Myristica fragrans* possesses holocentric chromosomes

**Fig. 1:**
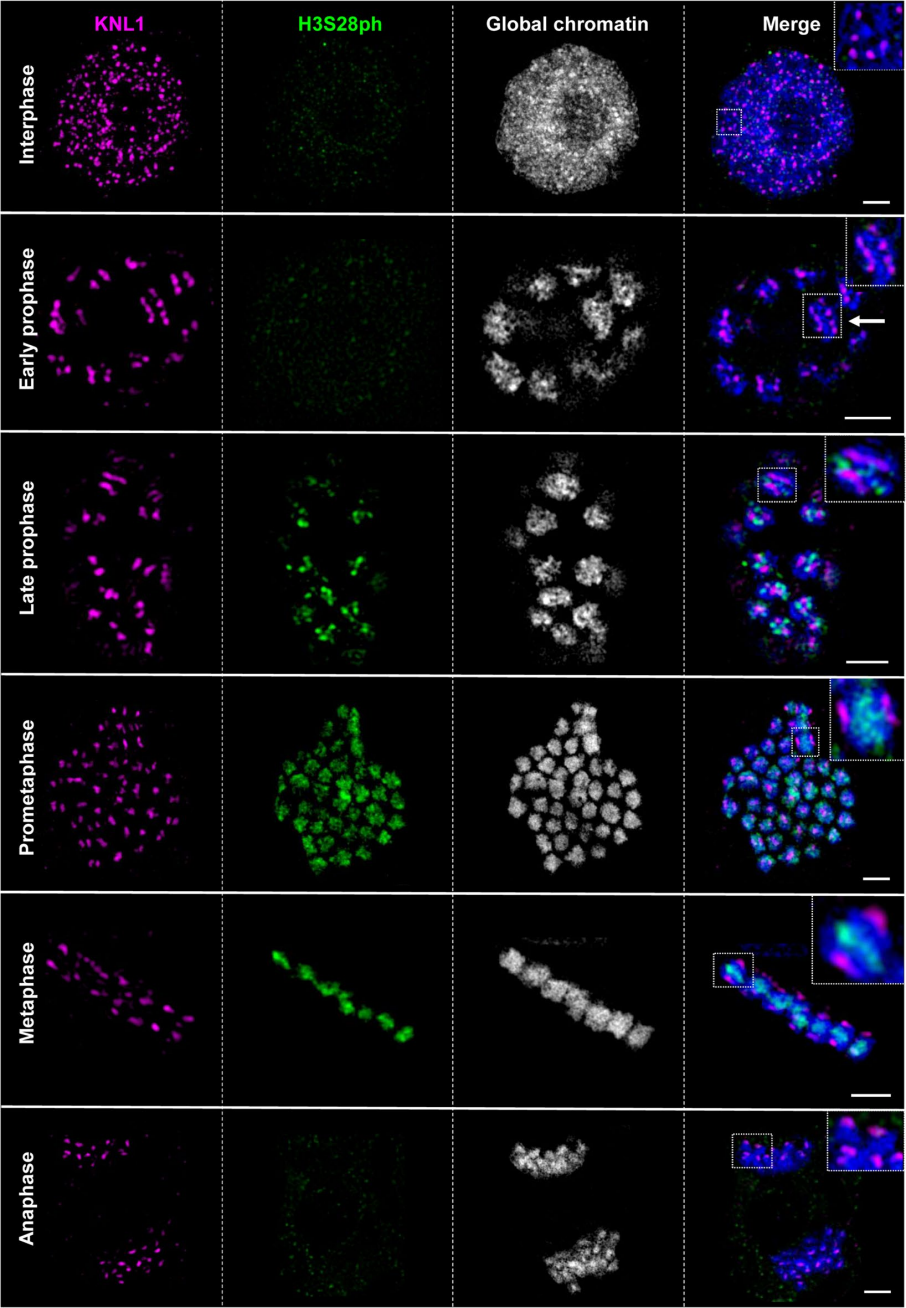
Localization and dynamics of KNL1 and H3S28 phosphorylation during the somatic cell cycle indicates holocentricity in *M. fragrans*. The ~300 KNL1 signals per interphase nucleus (see also Fig. 4b) associate during prophase, fuse to a line-like holocentromere at two poleward peripheries in prometaphase, and condense further in meta- and anaphase. The cell cycle-dependent H3S28 phosphorylation appears in late prophase, localizes within both chromatids and disappears after metaphase. All images represent single SIM slices. Only the interphase nucleus is displayed as a maximum intensity projection (MIP) to show all KNL1 signals inside. The magnified view of the respective region as demarcated by a dashed rectangle is shown in the inset. Global chromatin was counterstained by DAPI. Bars = 2 µm. Suppl. Movies 1, 2, 3, 4, 5, 6 visualize the 3D organization of all cell cycle stages at the super-resolution level based on 3D-SIM image stack rendering

The 1-2 µm long nearly isodiametric mitotic metaphase chromosomes of *Myristica fragrans* (2*n*=44, Suppl. Fig. 1) do not allow an unambiguous identification of the centromere type based solely on their chromosome morphology. A longitudinal centromeric groove as visible in other holocentric plants (e.g. *Luzula nivea* and *L. elegans* (Nagaki et al. 2005; Wanner et al. 2015) and *R. pubera* (Marques et al. 2015), reviewed in Schubert et al. (2020)) was not detectable. Therefore, first, we performed immunolabelling on mitotic cells for the conserved outer kinetochore protein KNL1 (Oliveira et al. 2024) and the cell-cycle dependent, (peri)centromere-enriched phosphorylated histone H3 serine 28 (H3S28ph) mark (Gernand et al. 2003). We then examined their localization using spatial structured illumination microscopy (3D-SIM). Anti-KNL1 marks the kinetochore in all stages of the mitotic cell cycle in a wide range of mono- and holocentric plant species (Neumann et al. 2023; Oliveira et al. 2024). In interphase nuclei, anti-KNL1 signals appeared dispersed within the chromatin. At early prophase, multiple dot-like KNL1 signals were found at two peripheries of the chromosomes. With further chromosome condensation at late prophase, the H3S28ph signals appeared, KNL1 signals fused and resulted in line-like signals. At metaphase, the H3S28ph signals were distributed along the entire length of chromosomes and restricted in between the pole-oriented KNL1 signals. After the separation of sister chromatids, H3S28ph signals disappeared at anaphase (Fig. 1; Suppl. Movies 1, 2, 3, 4, 5, 6).

**Fig. 2:**
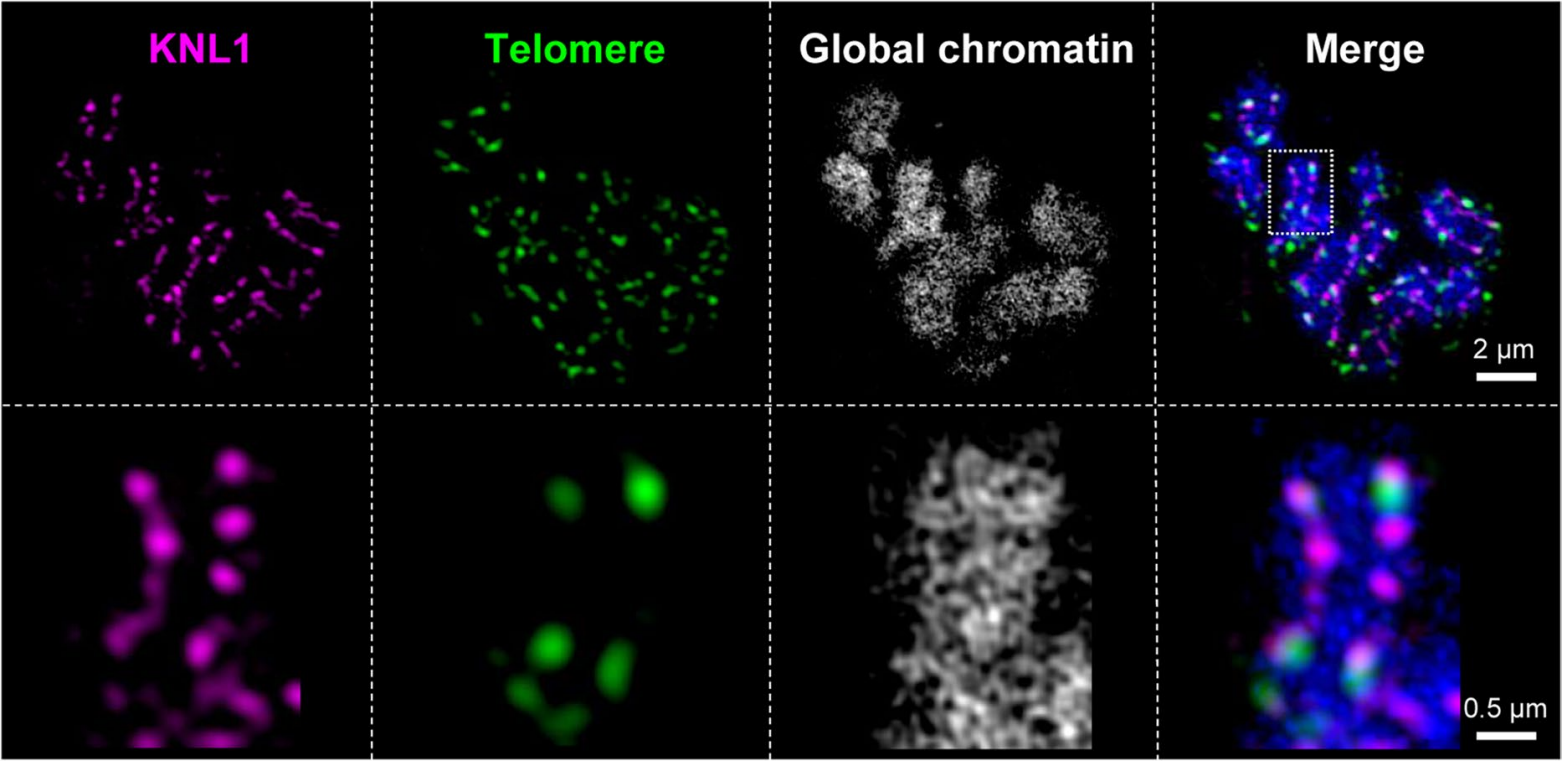
KNL1 and *Arabidopsis*-type telomere signals in somatic prometaphase chromosomes. The telomere signals localize at both ends of the dot line-like KNL1 signals. A magnified perspective of a chromosome as demarcated by a dashed rectangle is shown in the bottom panel. Maximum intensity projection of a 3D-SIM image stack

**Fig. 3:**
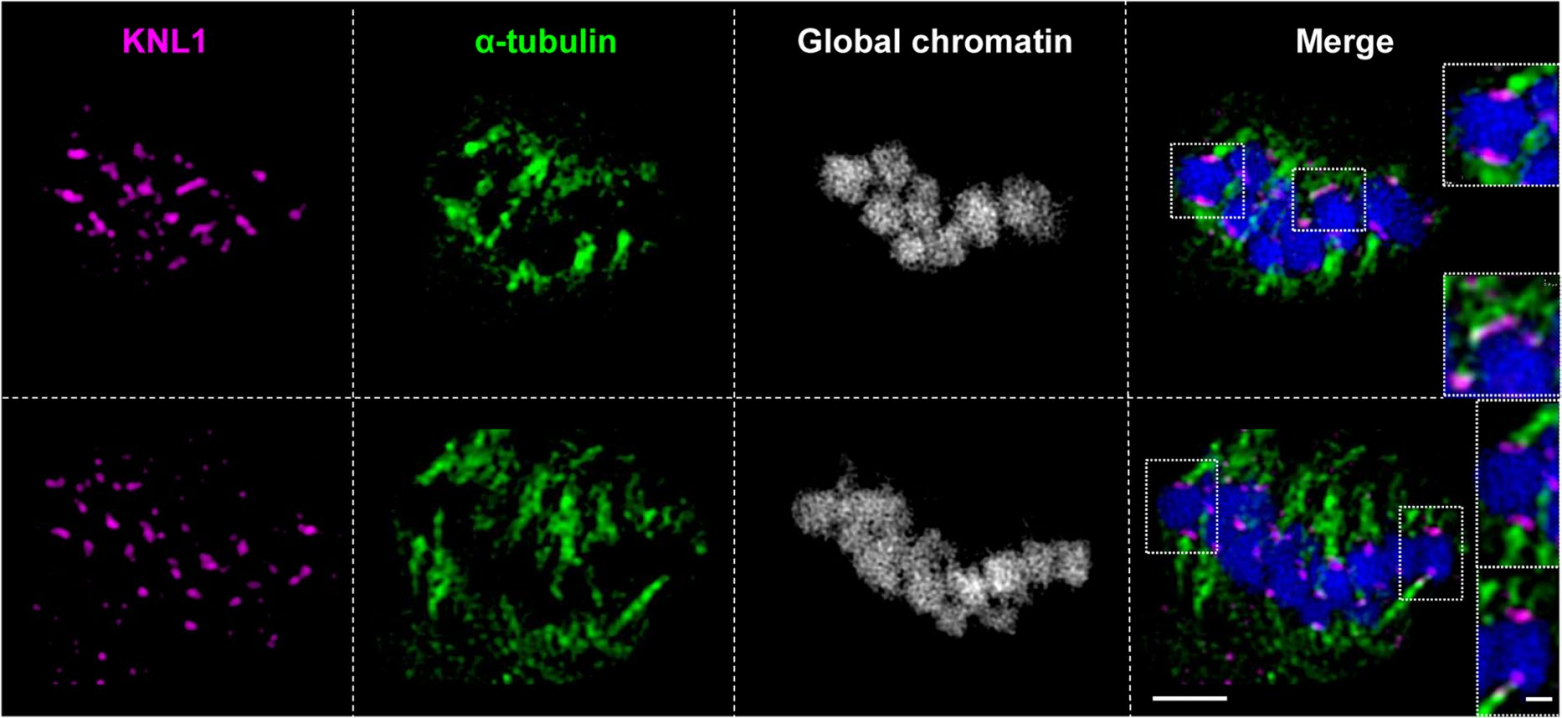
α**-**tubulin localizes to KNL1 signals indicating holocentricity in somatic metaphase chromosomes. The insets show α-tubulin attachment at enlarged chromosomes visible in two different slices of a 3D-SIM image stack visualized in Suppl. Movie 7. Suppl. Movie 8 shows the same cell based on 3D-SIM image stack rendering. Bar in whole cell image = 2 µm, in inset = 0.5 µm

To analyze the chromosome-wide distribution of KNL1 signals, we performed FISH using an *Arabidopsis*-type specific telomere probe on anti-KNL1 labelled prophase chromosomes. The presence of telomere signals at both ends of the line-like KNL1 signals confirmed a holocentromere-typical telomere-to-telomere distribution of KNL1 (Fig. 2). In addition, we observed colocalization of KNL1 signals along with mitotic spindle microtubule attachment sites throughout the entire length of the chromosomes (Fig. 3; Suppl. Movies 7, 8).

**Fig. 4:**
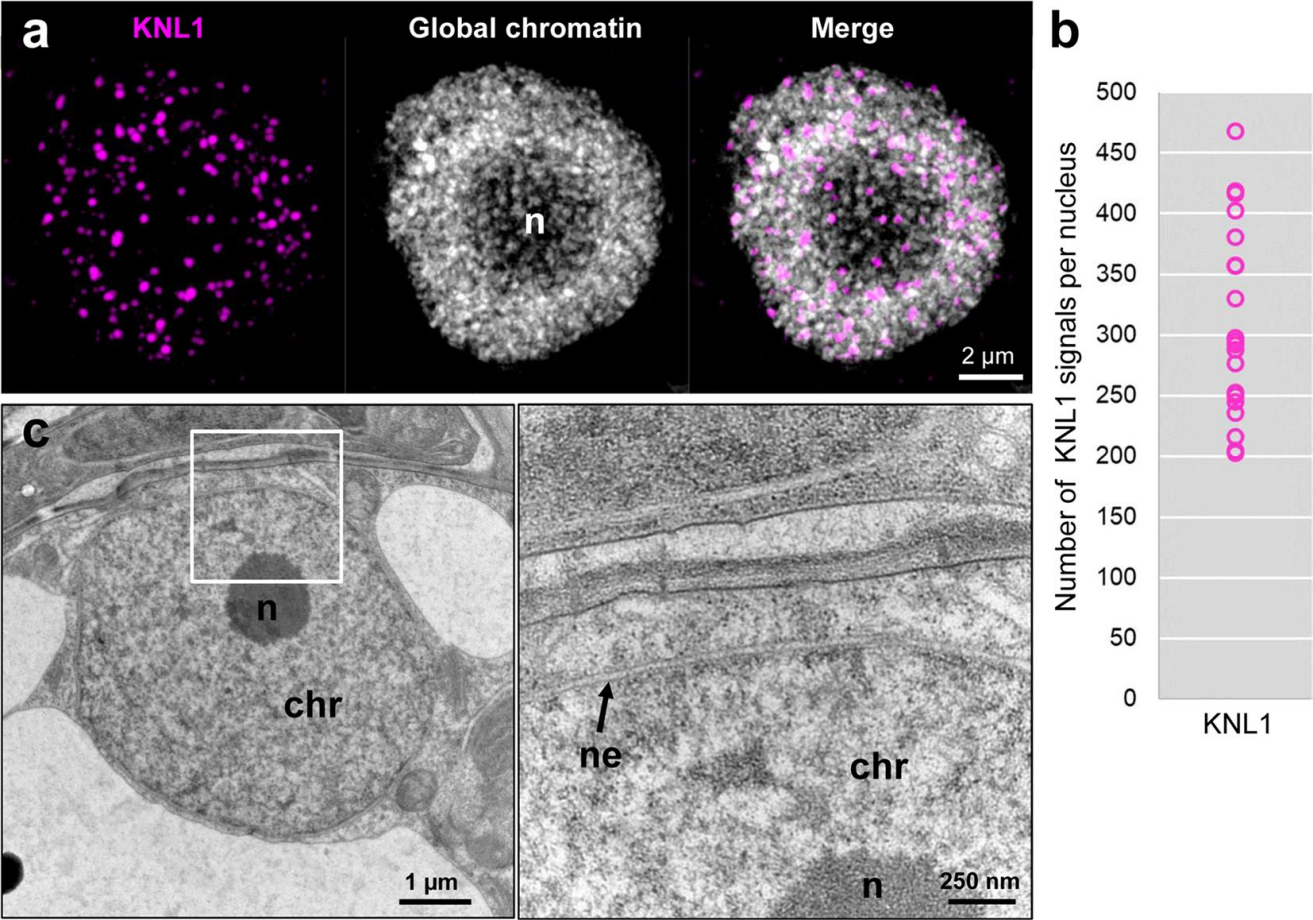
Super-resolution microscopy (3D-SIM) and transmission electron microscopy (TEM) indicates a dispersed chromatin arrangement without prominent chromocenters in interphase nuclei*.* (**a**) Around 245 KNL1 immuno signals, uniformly distributed within the DAPI-stained nucleus (global chromatin) were quantified using the Imaris 9.7 software tool “Spots”. Suppl. Movie 9 shows the same cell based on 3D-SIM image stack rendering. The nucleolus (n) appears less densely stained by DAPI. (**b**) Quantification of KNL1 signals from 3D-SIM image stacks of 20 interphase nuclei. (**c**) TEM of a leaf interphase nucleus confirms the dispersed chromatin (chr) distribution surrounded by the double-layered nuclear envelope (ne). The nucleolus (n) appears electron-dense. A magnified view of the region, marked in a rectangle, is shown on the right

The number of KNL1-immunofoci at interphase was counted as an additional feature to confirm the holocentricity. Due to the centromere unit-based composition of holocentromeres, the number of centromere-specific signals at interphase exceeds the number of chromosomes (Kuo et al. 2023). Therefore, we counted the number of KNL1 foci in 3D image stacks of 20 interphase nuclei (Fig. 4a, b, Suppl. Movie 9). The total number of KNL1 foci ranged from 203 to 468, much higher than the chromosome number of *M. fragrans* (2*n*=44). Considering the highest number as the maximal number of centromere units per nucleus and lower numbers as results of the association of several units, we conclude that each holocentromere per chromatid is likely composed of several centromere units, on average 10. However, we can’t exclude that the observed variation in signal number is partially caused by the cell cycle stage of the analyzed nuclei. In contrast to the holocentric plant *C. japonica*, which contains only a few centromere units per chromatid (Kuo et al. 2023), no prominent chromocenters were found via DAPI staining in *M. fragrans* nuclei (Figs. 1 and 4a). Further, transmission electron microscopy (TEM) also revealed the absence of prominent electron-dense regions in interphase chromatin (Fig. 4c). Thus, we conclude that *M. fragrans* is a holocentric species and its holocentromeres are composed of only a few centromere units which do not cluster and form chromocenters at interphase.

### Satellite repeats with the highest abundance do not represent the centromeric sequences

Holocentric species exist with and without centromere-specific repeats (reviewed in Schubert et al. (2020)). To determine whether *M. fragrans* possesses a repeat-based holocentromere, we analyzed the repeat composition of female and male plants. The existence of sex chromosomes in this species is uncertain (Flach 1966). Hence, we determined the genome size of male and female plants and applied paired-end genome sequencing to access sex-associated differences in the high-copy repeat composition.

**Fig. 5:**
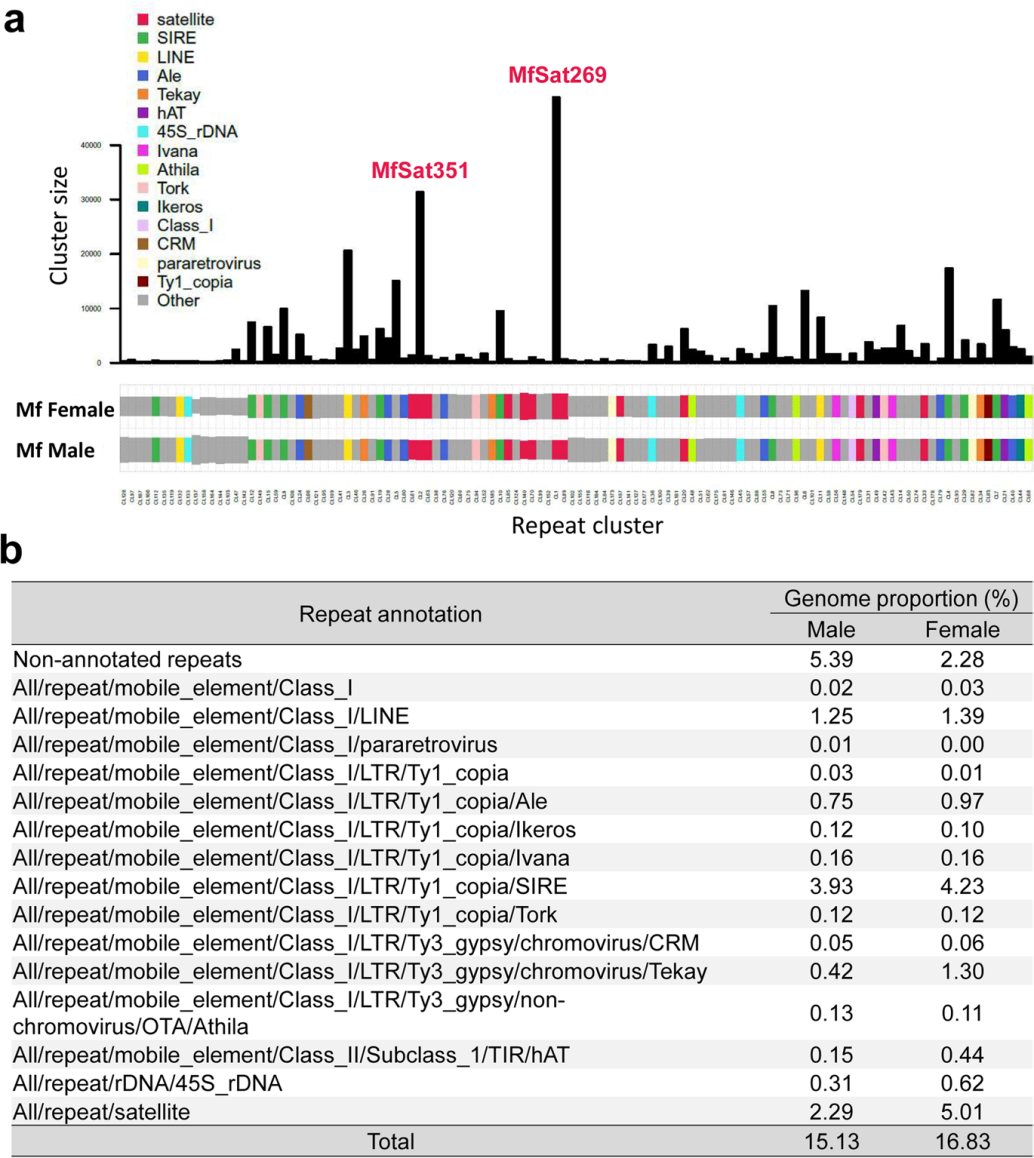
Genome-wide repeat analysis in *M. fragrans.* **(a)** Comparative analysis of repetitive genome fraction between male and female plants. The bar plot shows the size (number of reads) in each repeat cluster, and the colors represent the annotation of repeat types. **(b)** The proportion of moderate- and high-copy DNA repeats in the genomes of male and female plants

Flow cytometry analysis revealed that the three male plants examined displayed an average genome size of 701 Mbp/1C. Similarly, a lone female plant exhibited a similar genome size of 691 Mbp/1C (Suppl. Fig. 2). Next-generation sequence reads were generated to investigate the repeat composition of the genome by graph-based clustering analysis (Novak et al. 2017; Novák et al. 2020), resulting in the identification of high-copy satellite repeats and transposable elements (TEs). The comparative RepeatExplorer analysis indicated that all high-copy repeat clusters were shared and equally abundant in the male and female samples, and no sex-specific repeat cluster was found (Fig. 5a). The repeat proportion of the genome is relatively low, only 15.13% and 16.83% in the male and female samples, respectively (Fig. 5b). Among the annotated repeats, the Ty1 copia-SIRE retrotransposon is the most abundant repeat type in both samples, 3.93% and 4.23%, followed by satellite DNAs, 2.29% and 5.01%, and LINEs (long interspersed elements), 1.25% and 1.39%, respectively. The genome proportion of the other annotated repeat types is less than 1%. Hence, there appears to be no severe difference in the repeat composition between male and female *M. fragrans*. Consequently, the likelihood of having repeat-enriched heteromorphic sex chromosomes is low.

**Fig. 6:**
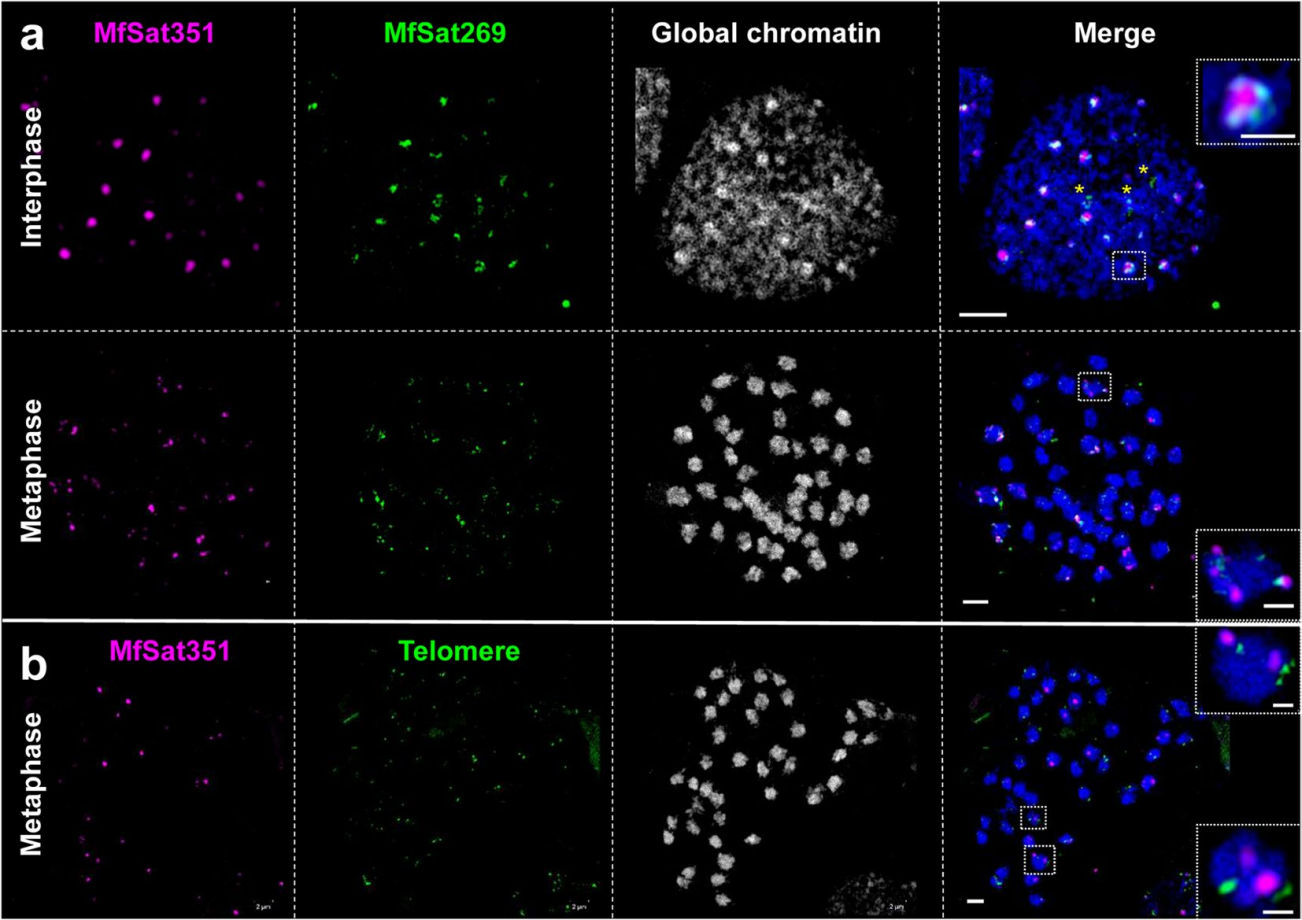
The most abundant satellite repeats MfSat351 and MfSat269 do not constitute the holocentromeres. (**a**) In interphase, while MfSat351 always colocalizes to MfSat269, MFSat269 also localize independently of MfSat351 (asterisks). In metaphase, the repeats display varying degrees of visibility, ranging from being absent to abundant among chromosomes, which suggests that they are not associated with the centromeres. (**b**) Metaphase chromosomes showing the localization of MfSat351 and telomeres. Enlarged regions are marked by dashed rectangles. All images represent single SIM slices. Bars in whole cell images = 2 µm, in inset = 0.5 µm

Since satellite repeats with the highest genome proportion are often centromere-specific (Plohl et al. 2014), we checked the chromosomal distribution of the two most abundant high-copy satellites MfSat269 (monomer length 269 bp) and MfSat351 (monomer length 351 bp) (Fig. 5a). After FISH, neither MfSat269 nor MfSat351 displayed line-like signals in metaphase chromosomes, characteristic of holocentric chromosomes (Fig. 6). In contrast, MfSat351 and MfSat269 are often localized next to each other in subtelomeric positions (Fig. 6a). The subtelomeric position of most MfSat351 sites was further confirmed by cohybridization with an *Arabidopsis*-type telomere-specific probe (Fig. 6b). Thus, we conclude that the most abundant high-copy satellite repeats MfSat351 and MfSat269 in the *M. fragrans* genome do not represent centromeric repeats.

## Discussion

**Fig. 7:**
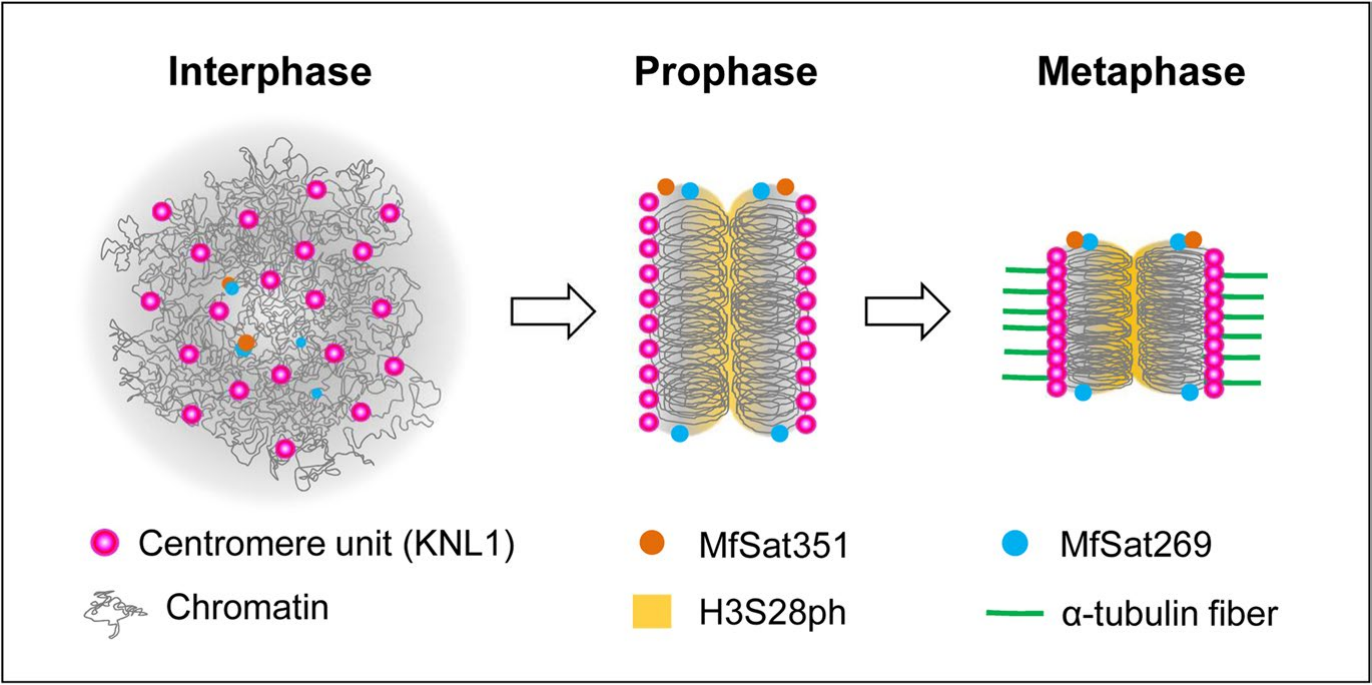
Model depicting the mitotic dynamics of *M. fragrans* holocentromeres. Each KNL1 signal represents a centromere unit. Each holocentromere comprises, on average, 10 non-satellite DNA-based centromere units, scattered in the interphase nucleus. Through chromatin folding, at prophase, the centromere units align at two poleward peripheries of chromosomes at the sites of microtubule attachment. At metaphase, the stage at which the chromosome is at its maximum condensation, the scattered centromere units coalesce to form a line-like holocentromere, at where microtubules attach. The cell cycle-dependent H3S28ph signals appear at prophase and highlight the pericentromeric regions of a chromosome. The two most abundant satellite repeats, MfSat351 and MfSat269, accumulate at chromosome ends, not associating with the holocentromeres. To simplify, only one chromosome is depicted

The small-sized mitotic chromosomes of *M. fragrans* are holocentric, as evidenced by the line-like distribution of KNL1, the signals of H3S28ph and tubulin in mitotic metaphase chromosomes, and by the presence of a much higher number of interphase centromere-unit signals than the chromosomes. The average number of 10 centromere units per *M. fragrans* holocentromere is comparable with the number of centromere units of the holocentric plants *C. japonica* (Kuo et al. 2023) and *Morus notabilis* (Ma et al. 2023). However, unlike *C. japonica*, *M. fragrans* centromere units do not form chromocenters during interphase, most likely due to the lack of centromeric satellite DNA and the low amount of heterochromatin (Fig. 7).

The centromere DNA composition across independently evolved holocentrics is diverse, and *M. fragrans* adds an example of a non-major satellite-based holocentromere species. In other species, the CENH3-based holocentromeres can either associate with particular satellite DNAs and/or transposable elements (TEs), as in the nematode *Meloidogyne* (Slade et al. 2021), the plants *C. japonica* (Kuo et al. 2023), *Rhynchospora* species (Marques et al. 2015; Hofstatter et al. 2022; Castellani et al. 2024) and *Cuscuta europaea* (Oliveira et al. 2020), or show no sequence specificity as in the nematode *Caenorhabditis elegans* (Gassmann et al. 2012) and the plant *L. elegans* (Heckmann et al. 2013) (Table 1). The fact that the satellite repeats with the highest proportion in the genome did not show holocentromere-like FISH signals opens the possibility that *M. fragrans* might possess transposable elements or only epigenetically defined centromere units. In addition, the total repetitive fraction of the *M. fragrans* genome, ~17%, is relatively low in comparison to other plant species having a similar small genome size (Novak et al. 2020). In this dioecious species, neither a significant difference in genome size (*P*=0.14; Mann-Whitney Rank Sum test) exists between male and female individuals, nor severe detectable sex-specific accumulation of repeat DNA. Therefore, it is not very likely that heteromorphic sex chromosomes exist in *M. fragrans*. However, our analysis of high- and medium-copy repeats does not exclude the existence of minor sex-specific repeats in the male and female genomes of *M. fragrans*.

**Table 1:** The presence and absence of centromeric nucleosomes and DNAs in the holocentric species

Species	CENH3/CENPA-based units	Centromeric satellites (monomer size)	Centromeric TEs (TE type)	References
*Caenorhabditis elegans*	Yes	No	No	(Buchwitz et al. 1999; Gassmann et al. 2012)
*Meloidogyne incognita*	Yes	Yes (45-83 bp)	No	(Slade et al. 2021)
*Bombyx mori*	No	No	No	(Senaratne et al. 2021; Drinnenberg et al. 2014)
*Luzula elegans*	Yes	No	No	(Heckmann et al. 2013)
*Rhynchospora pubera*	Yes	Yes (*Tyba*, 172 bp)	Yes (Ty3/gypsy)	(Marques et al. 2015; Hofstatter et al. 2022)
*Cuscuta europaea*	Yes*	Yes (CUS-TR24, 389 bp)	No	(Oliveira et al. 2020; Neumann et al. 2023)
*Morus notabilis*	Yes	Yes (*m3cp*, 82 bp)	No	(Ma et al. 2023)
*Chionographis japonica*	Yes	Yes (*Chio*, 23 and 28 bp)	No	(Kuo et al. 2023)

^*^The CENH3 of *C. europaea* either lost its centromere function or acts in parallel to an additional CENH3-independent mechanism of kinetochore assembly (Neumann et al. 2023; Oliveira et al. 2020)

The genus *Myristica* belongs to the Myristicaceae family, the clade Magnoliids, which is the third largest group of flowering plants after monocots and eudicots. The Myristicaceae family consists of about 520 species, classified into 21 genera, and is widely distributed across Asia, Africa, and America (Li and Wilson 2008). The confirmation of *M. fragrans* as a holocentric species brings forth an opportunity to study the distribution and evolution of holocentric chromosomes in the clade Magnoliids. To further unveil the holocentromere organization in *M. fragrans*, advanced genome assembly methods and the generation of *Myristica*-specific CENH3 antibodies for subsequent CENH3-ChIPseq are necessary.

### Supplementary Information

Below is the link to the electronic supplementary material.

Supplementary file1 (TIF 3184 KB)

Supplementary file2 (TIF 3318 KB)

Supplementary file3 (MP4 31.4 MB)

Supplementary file4 (MP4 24.8 MB)

Supplementary file5 (MP4 25.2 MB)

Supplementary file6 (MP4 32.7 MB)

Supplementary file7 (MP4 25.0 MB)

Supplementary file8 (MP4 13.8 MB)

Supplementary file9 (AVI 10.9 MB)

Supplementary file10 (MP4 42.6 MB)

Supplementary file11 (MP4 38.2 MB)

## Data Availability

The sequence datasets that used for repeatome analysis are available on request from the corresponding author.
