# Supplementary figures and images for: The holocentricity in the dioecious nutmeg (Myristica fragrans) is not based on major satellite repeats
Source: Chromosome Res. 2024 May 8;32(2):8. doi: 10.1007/s10577-024-09751-1 (PMC11078807; doi:10.1007/s10577-024-09751-1)

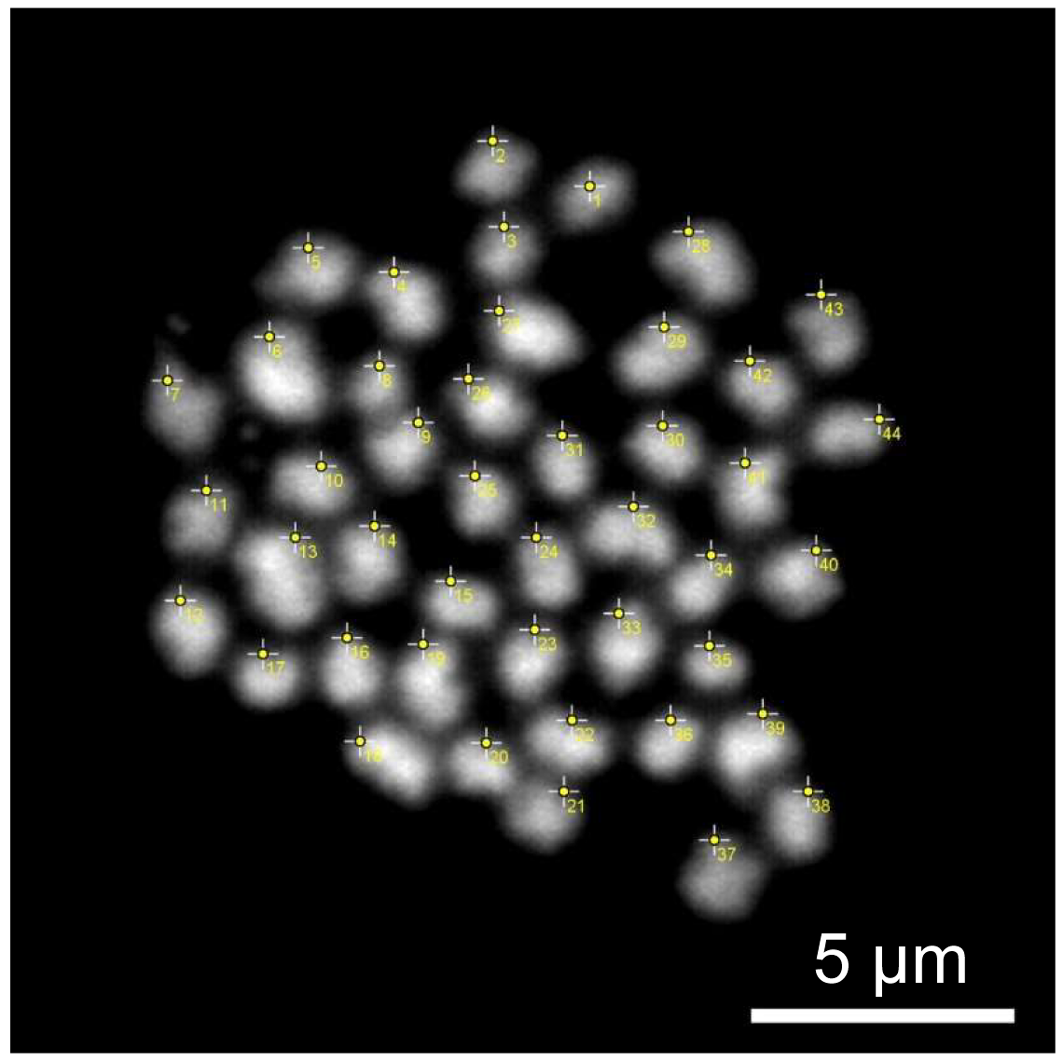

Supplement: Supplementary file 1 — Supplementary file1 (TIF 3184 KB) [file 10577_2024_9751_MOESM1_ESM.tif]

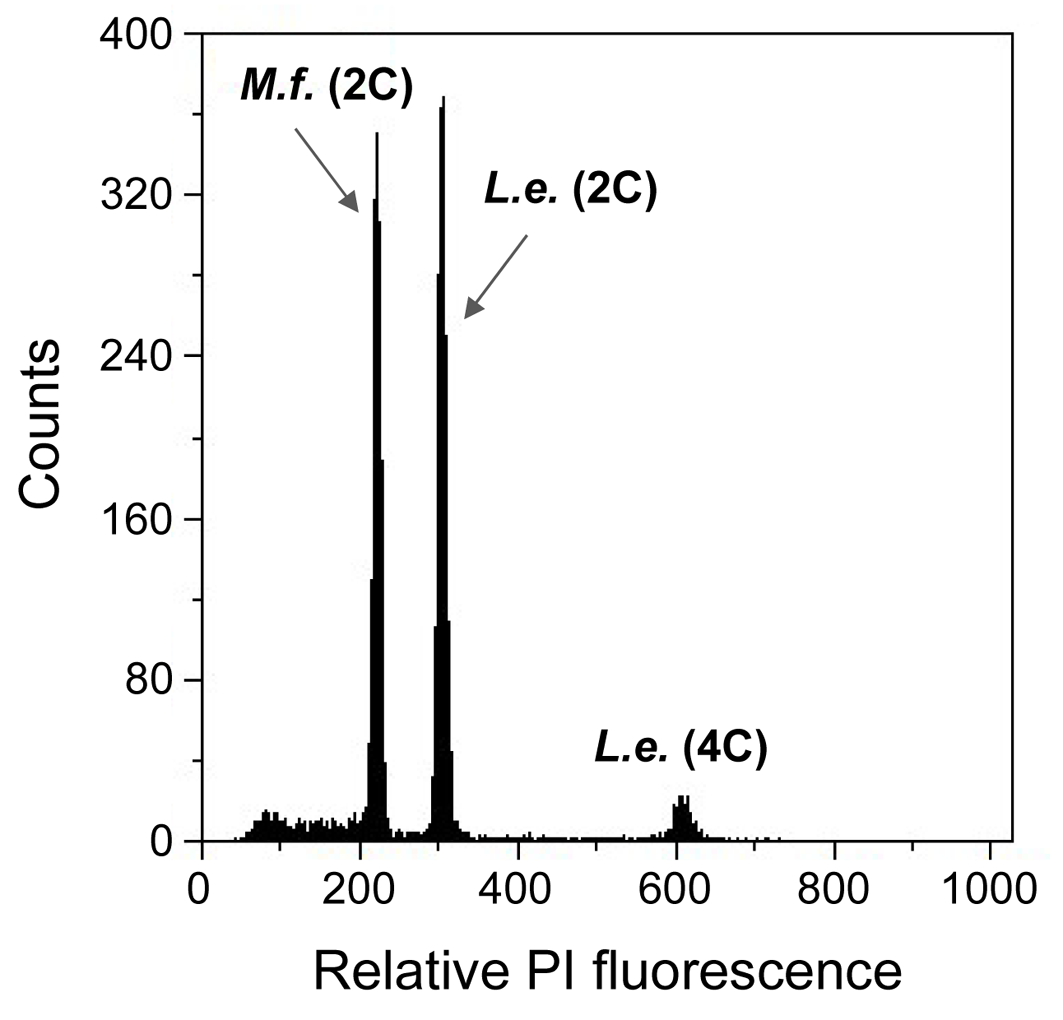

Supplement: Supplementary file 2 — Supplementary file2 (TIF 3318 KB) [file 10577_2024_9751_MOESM2_ESM.tif]
